# Supplementary material for: The discovery of novel noncoding RNAs in 50 bacterial genomes
Source: Nucleic Acids Res. 2024 Apr 22;52(9):5152–65. doi: 10.1093/nar/gkae248 (PMC11109978; doi:10.1093/nar/gkae248)
Supplement: gkae248_Supplemental_Files [file gkae248_supplemental_files.zip › Narunsky 50 Genomes RNA Motifs Files.zip!/Publication/46-B_bacilliformis/Figures/LRC-46-3-NC_008783_1_IGR_8_223845_224044.sto.pdf]

LRC-46-3-NC_008783_1_IGR_8_223845_224044.sto.r2r

5´-UCAGUUG●●G●AR○●RYA●UAUU●U●A●C●●RRRY●RUUG●AAARRURRCCGCU●UAU●CY●●AAUUUUUGAAAAAUCCUAAUCAGUAAAAAACAACUGAUUGCAAGGCAAAAAUACUUAAGCUCUAUGACAUAUUUGAAUUGCAUACUUUUCCAUAAAUCCCCYCCCCUU●UAUUUUUUAAUCCUG
